# Climate Change and Infectious Diseases: Navigating the Intersection through Innovation and Interdisciplinary Approaches

**DOI:** 10.3390/ijerph21030314

**Published:** 2024-03-08

**Authors:** Prisco Piscitelli, Alessandro Miani

**Affiliations:** 1 Italian Society of Environmental Medicine (SIMA), Viale di Porta Vercellina, 9, 20123 Milan, Italy; 2 Department of Experimental Medicine, University of Salento, Via Monteroni, 73100 Lecce, Italy; 3 Environmental Health Research Organization (EHRO), San Diego, CA 92123, USA

The era of climate change has introduced unprecedented challenges for global public health, especially visible through the lens of infectious diseases [1,2]. As the planet warms, the delicate balance between ecosystems and pathogens shifts, leading to the emergence and re-emergence of infectious diseases. Scientists and researchers must unravel the intricate dynamics between climate change and infectious diseases due to the urgent need for integrated research, innovative technologies, and robust public health strategies to mitigate these risks.

Climate change, as manifested through global warming and shifts in weather patterns, is reshaping the landscape of public health, particularly for infectious diseases [3]. Changes in temperature and precipitation levels and the frequency and intensity of extreme weather events have profound implications for the distribution and behavior of disease vectors, such as mosquitoes, ticks, and rodents [4,5]. These changes, observable and measurable, directly influence vectors’ life cycles, breeding patterns, and migration routes, thereby affecting the transmission dynamics of the diseases that they carry.

Temperature plays a critical role in vector biology, influencing metabolic rates and the speed of pathogen development within vectors. Increased temperatures can accelerate the mosquitoes’ life cycles, such as by shortening the time required for a mosquito to develop from egg to adult, thereby increasing the population density of mosquitoes in a given area [3]. This acceleration also reduces the incubation period of pathogens such as the malaria parasite (*Plasmodium* spp.) and the dengue virus within mosquitoes, increasing the transmission potential [6]. Consequently, regions previously deemed too cold for vector survival and pathogen development now face emerging threats as global temperatures rise.

Precipitation patterns also significantly impact vector-borne disease dynamics [7]. Increased rainfall can create ideal breeding sites for mosquitoes through standing water accumulation, while drought conditions compel both humans and vectors to share diminishing water sources, increasing contact rates and the disease transmission potential. The complexity of these interactions underscores the variability in climate change impacts across different regions and diseases. For example, while some areas may experience a surge in mosquito populations following heavy rains, others may witness vector populations reductions due to drought-induced breeding site scarcity [7].

Extreme weather events, such as hurricanes and floods, can further exacerbate the situation by disrupting existing public health infrastructure, creating new breeding grounds for vectors, and displacing populations, thereby facilitating the spread of infectious diseases. The aftermath of such events often results in spikes in diseases like malaria, dengue fever, and cholera, emphasizing the need for robust disaster preparedness and response strategies that incorporate infectious disease management.

The expansion of mosquito-borne diseases, such as malaria and dengue fever, into new geographical areas starkly illustrates climate change’s impact on infectious disease patterns [6]. As temperatures rise, the geographic range of mosquitoes that carry these diseases expands, introducing these pathogens to previously unaffected regions and populations. This shift not only poses a direct threat to public health but also challenges existing healthcare systems and disease control measures, which may not be equipped to deal with these emerging threats.

Rainfall pattern changes affect the availability and quality of water, influencing not only vector-borne diseases but also water-borne diseases such as cholera and leptospirosis. The interplay between these factors illustrates the complex and multifaceted nature of the relationship between climate change and infectious diseases, highlighting the urgent need for interdisciplinary approaches to understand, predict, and mitigate these emerging health risks.

Environmental photocatalysis, a cutting-edge technology that harnesses light to initiate chemical reactions, is a vital innovation in the ongoing battle against climate change-exacerbated pathogen transmission. This approach uses light-activated catalysts, such as titanium dioxide (TiO_2_) and its composite materials, among the most effective and widely researched examples. These catalysts can absorb ultraviolet (UV) light and use this energy to drive chemical reactions to decompose organic pollutants and pathogens present in water and air, rendering them harmless [8,9,10,11,12,13,14,15,16]. This technology not only promises a sustainable method for purifying environmental mediums but is also a potent tool for reducing the vectors of disease that thrive under polluted conditions.

Photocatalysis’ underlying principles depend on the catalytic properties of materials like titanium dioxide. When these materials are exposed to UV light, they generate electron-hole pairs that can interact with water and oxygen molecules, producing reactive oxygen species (ROS) [17,18,19]. These ROS, including hydroxyl radicals and superoxide anions, are highly reactive and can effectively break down various organic compounds and microorganisms. This reaction does not alter the catalyst, meaning that titanium dioxide can be used repeatedly without losing its efficacy, making it an environmentally friendly and cost-effective solution for long-term use.

Regarding public health, environmental photocatalysis has particularly promising water and air purification applications. For water purification, photocatalytic processes can degrade harmful pathogens and pollutants, creating safe drinking water and reducing the risk of waterborne diseases, crucial in areas affected by climate change-induced alterations in water quality and availability. This technology can be applied in both large-scale water treatment facilities and small-scale portable water purification systems, making it a flexible solution for addressing diverse needs and contexts.

For air purification, photocatalytic systems can be integrated into HVAC systems [20] or used in standalone air purifiers to remove airborne pathogens and pollutants, including volatile organic compounds (VOCs) and particulate matter [21]. This fact is particularly relevant in urban and industrial areas, where air quality is a significant public health concern. By reducing airborne pathogen concentrations, photocatalysis is crucial for preventing the spread of respiratory diseases, countering increased transmission rates caused by climate change.

Integrating photocatalytic materials into building materials, such as paints and coatings, is an innovative approach to creating self-cleaning and air-purifying surfaces [17]. Crucially, nanotechnologies also have the potential to significantly reduce CO_2_ emissions associated with traditional building materials production [22], thereby addressing a key driver of climate change. This application not only creates healthier indoor environments but also mitigates outdoor pollution, offering a dual benefit in fighting climate-induced health risks.

Despite its potential, the widespread adoption of environmental photocatalysis faces challenges, including the need for UV light, limiting its applicability in low-light conditions. However, ongoing studies are focusing on developing catalysts that operate under visible light, broadening the scope of this technology’s use. 

Additionally, optimizing the efficiency, durability, and cost-effectiveness of photocatalytic materials will allow for their extensive use in public health applications.

Environmental photocatalysis is a forward-thinking solution that can resolve the intertwined challenges of pollution, pathogen transmission, and climate change. By leveraging the power of light-activated catalysts for water and air purification, this technology is promising for enhancing environmental health and reducing the disease burden. As research advances and applications expand, environmental photocatalysis could become a cornerstone of sustainable public health strategies in response to a climate change.

The advent of graphene-based nanomaterials heralds a transformative era in environmental remediation [23,24,25], particularly for mitigating the impacts of climate-sensitive infectious diseases. Graphene, a single layer of carbon atoms arranged in a two-dimensional honeycomb lattice, has remarkable properties that make it ideal for purifying water and air. Its exceptionally high surface area, coupled with its reactivity and conductivity, allows graphene to efficiently adsorb and breakdown various contaminants, including pathogens, heavy metals, and organic pollutants.

Graphene-based materials use several mechanisms to purify environmental media. In water remediation [25], these nanomaterials adsorb toxic substances onto their surfaces, thus removing them from the water. Furthermore, modified graphene oxide, with functional groups attached to its surface, targets specific pollutants through chemical reactions, degrading or transforming them into less harmful substances. This specificity is crucial for combatting the varied contaminants that threaten water quality and, consequently, public health.

In air purification, graphene-based filters capture particulate matter, volatile organic compounds (VOCs), and pathogens, including viruses and bacteria. The material’s high conductivity [26] means that it can potentially inactivate captured microbes through electrical or thermal processes, adding an extra layer of protection against airborne diseases. This capability is particularly significant in urban and industrial settings, where air quality is a persistent concern, and healthcare environments, where preventing the spread of infectious diseases is vital.

Graphene-based nanomaterials have more benefits than their efficacy at removing pollutants [24]. Their versatility allows for their integration into various platforms, from standalone water filters and air purifiers to coatings and membranes applicable to existing infrastructure. This adaptability ensures that graphene-based solutions can be tailored to specific environmental and public health needs, offering a scalable approach for combatting climate-sensitive infectious diseases’ spread.

However, despite their promising potential, several challenges must be addressed before we can fully harness graphene-based nanomaterials’ capabilities in environmental health. One of the primary concerns is production costs and scalability. While graphene has exceptional properties, producing it in forms suitable for large-scale environmental applications is expensive. Research and development efforts are ongoing to identify cost-effective and sustainable methods for producing graphene and incorporating it into practical applications.

Another challenge involves understanding the long-term environmental and health impacts of graphene-based materials [27]. As with any nanomaterial, researchers need to conduct comprehensive studies to assess the potential risks associated with graphene’s widespread use, including toxicity and environmental persistence [28]. Ensuring graphene-based technologies’ safe deployment requires rigorous testing and regulatory oversight to prevent unintended consequences.

Developing graphene-based nanomaterials for environmental remediation makes it easier for us to address the complex challenges posed by climate-sensitive infectious diseases. By leveraging the unique properties of graphene, we can develop innovative solutions to purify water and air, thereby reducing pathogen and pollutant transmission. As we continue to explore the full potential of these nanomaterials, addressing the challenges of cost, scalability, and safety will be crucial for realizing their promise for environmental health and disease prevention.

Machine learning algorithms represent a key predictive health innovation, as they convert vast and complex datasets into actionable insights [29]. These advanced computational tools assess the intricate interplay between climate variables and the dynamics of infectious disease spread, offering a robust framework for modeling and forecasting future outbreaks [30]. By using data-driven models, researchers and public health officials can identify emerging of infectious disease patterns and hotspots, facilitating the implementation of preventative measures and timely interventions [31].

Machine learning learns from historical data, continuously improving its predictive accuracy over time. Algorithms can analyze trends related to temperature, humidity, rainfall, and other environmental factors, alongside historical disease incidence rates, to forecast future outbreaks. This predictive capability is invaluable given that climate change is altering traditional disease vectors and transmission patterns, making historical data less indicative of future trends.

One illustrative case study applied machine learning algorithms to predict dengue fever outbreaks [32]. The researchers utilized climate data, including temperature and precipitation patterns, in conjunction with socio-economic and demographic information, to identify regions at increased risk of dengue transmission. These models have enabled public health authorities to more effectively allocate resources, targeting mosquito control efforts and public health campaigns in high-risk areas before the peak transmission season begins [33].

Another study used machine learning to forecast the spread of influenza [34]. By analyzing data from a variety of sources, including health records, search engine queries, and climate information, algorithms were used predict the onset and intensity of flu seasons with remarkable accuracy. This approach improved preparedness among healthcare providers and the public, enabling vaccinations and other preventive measures to mitigate the flu season’s impact.

These case studies’ success underscores the transformative potential of integrating machine learning algorithms into public health planning and response strategies. However, these tools’ effective application requires high-quality, comprehensive datasets, as well as interdisciplinary collaboration between epidemiologists, climate scientists, data scientists, and public health professionals. Together, these experts can refine predictive models, validate their forecasts against real-world outcomes, and adapt strategies that reflect the evolving landscape of infectious disease risks.

Machine learning algorithms are also critical for enhancing disease surveillance systems. By integrating real-time data from a variety of sources, including social media and electronic health records, these algorithms detect early signs of an outbreak, decreasing faster response times and potentially saving lives.

The use of machine learning in predictive health is a significant advancement in our ability to combat infectious diseases in response to climate change. By identifying patterns and predicting outbreaks, these algorithms are a critical tool for enabling timely public health interventions. As technology advances and our understanding of the complex relationship between climate variables and disease spread deepens, integrating machine learning into public health strategies will become increasingly indispensable.

The intricate relationship between climate change and infectious disease incidence is significantly influenced not only by direct climatic factors such as temperature and precipitation but also by various non-climate factors. These factors include social vulnerability, the immune statuses of populations, and the efficacy of vector control efforts [35]. Understanding the interplay between these elements is vital for the comprehensive assessment and effective management of infectious disease risks. This nuanced approach acknowledges that climate change’s impact on health is mediated by complex socio-economic and biological variables that either exacerbate or mitigate disease transmission.

Social vulnerability, encompassing the capacity of communities to anticipate, cope with, respond to, and recover from the health-related impacts of climate change, is critical in infectious disease dynamics [36]. Factors such as poverty, a lack of access to healthcare, inadequate housing, and high population density can amplify the risk of disease outbreaks. These conditions often correlate with limited resources being available for disease surveillance and control, making certain populations more susceptible to infections’ spread following climatic events like floods or heatwaves. Understanding these vulnerabilities’ distribution can help with tailoring public health interventions to protect the most affected communities.

Immune status, another crucial non-climate factor, determines the susceptibility of individuals and populations to infectious diseases. Malnutrition, existing health conditions, and age are key immune competence determinants. Climate change indirectly influences these determinants by affecting food security, air quality, and the prevalence of diseases that compromise immune function. For instance, increased CO_2_ levels can lower staple crops’ nutritional quality, potentially weakening a population’s immunity and increasing its susceptibility to infections [37].

Vector control efforts, crucial in the fight against diseases like malaria, dengue fever, and Zika, are directly impacted by climate variables. Warmer temperatures and altered precipitation patterns can expand vector habitats, necessitating more extensive and sustained control efforts [36]. However, the effectiveness of these efforts is also contingent on non-climate factors, such as public awareness, community participation, and the availability of resources for implementing control measures. Strategies sensitive to local climatic and socio-economic conditions are more likely to reduce disease transmission.

Furthermore, the climate change’s secondary effects, such as natural disasters, present additional challenges for infectious disease management. Events such as hurricanes, floods, and droughts can displace populations, creating conditions ripe for disease outbreaks. Crowded temporary shelters, compromised water and sanitation systems, and disrupted healthcare services can facilitate the spread of infectious diseases among displaced populations. Additionally, the psychological stress associated with these events can weaken immune responses, further increasing vulnerability to infections.

Understanding the interplay between climate and non-climate factors is, therefore, not only an academic exercise but also vital for effective public health planning and interventions. It requires an integrated approach that combines climatic data with socio-economic and health indicators to accurately predict disease outbreaks and implement timely measures to mitigate their impact. By considering the full spectrum of factors influencing disease dynamics, public health authorities can develop more resilient and adaptive strategies to protect communities affected by changing climates.

A critical examination of interventions designed to mitigate the health risks associated with climate change must be performed. This intervention would involve conducting a multifaceted analysis of both the cost-effectiveness and overall efficacy of these programs. A thorough evaluation process is vital, as it would not only validate the interventions’ success in real-world settings but also ensure the optimal allocation of resources. Methodologies for this assessment range from quantitative analyses, such as cost-benefit and cost-effectiveness studies, to qualitative approaches that consider programmatic adaptability, scalability, and sustainability. These evaluations provide invaluable insights, helping policymakers and stakeholders to make informed decisions when implementing and scaling health interventions in response to climate change.

In assessing cost-effectiveness, analysts compare the intervention’s costs relative to its health outcomes, typically measured as quality-adjusted life years (QALYs) saved or infections averted. This analysis helps us to identify strategies with the greatest health impacts per unit of investment, an essential consideration in resource-limited scenarios. Program efficacy, on the other hand, is evaluated through metrics such as reductions in disease incidence, improvements in public health infrastructure, and enhanced community resilience. Longitudinal studies and post-implementation reviews are crucial for understanding these interventions’ sustained impacts of over time.

However, the complex nature of climate-sensitive health risks necessitate a broader perspective. 

The interaction between environmental changes and public health is influenced by a myriad of factors, including socio-economic conditions, urbanization patterns, and global mobility. As such, evaluating interventions requires an interdisciplinary approach that synthesizes data from diverse fields. Collaboration between epidemiologists, climatologists, urban planners, and social scientists is crucial to comprehensively assess climate change’s multifaceted impacts on health and devise effective and equitable strategies.

The urgency of the situation requires a proactive stance, leveraging the latest advancements in empirical research, theoretical modeling, and technological innovation. Digital health technologies, geographic information systems (GIS), remote sensing, and artificial intelligence (AI) are promising tools for monitoring disease vectors, predicting outbreaks, and optimizing resource deployment. These innovations, coupled with traditional public health measures and community engagement, are the cornerstone of an effective response to climate change-related health challenges.

In conclusions, the need for a coordinated, multidisciplinary approach for combatting climate-sensitive health risks cannot be overstated. These challenges’ complexity demands a concerted effort that brings together scientific inquiry, technological innovation, and policy formulation. By fostering collaboration between disciplines and sectors, we can develop robust, adaptable strategies that not only mitigate the immediate impacts of climate change on health but also build long-term resilience in vulnerable populations. This call to action underscores our collective responsibility to safeguard public health in response to an uncertain future, and we urge all stakeholders to unite to formulate a comprehensive response to climate change.
